# The gut-heart axis: a review of the mechanisms and therapeutic prospects of gut microbiota and their metabolites in cardiovascular disease

**DOI:** 10.3389/fcvm.2026.1909679

**Published:** 2026-08-03

**Authors:** Zhen-ni Yu, Xing-shun Zhou, Lu Yang, Yan Luan, Tai-biao Geng, Rui Wang, Run-jun Zhang, Cong Huang

**Affiliations:** 1 Department of Radiology, No. 926 Hospital, Joint Logistics Support Force of PLA, Kaiyuan, Yunnan, China; 2 Department of Nursing, No. 926 Hospital, Joint Logistics Support Force of PLA, Kaiyuan, Yunnan, China; 3 Department of Cardiology, No. 926 Hospital, Joint Logistics Support Force of PLA, Kaiyuan, Yunnan, China

**Keywords:** cardiovascular disease, gut microbiota, gut-heart axis, microbial metabolites, microbiome therapy, short-chain fatty acids, trimethylamine N-oxide

## Abstract

The gut microbiota, a vital virtual organ, communicates bidirectionally with the host cardiovascular system via its intricate metabolic network, forming the gut-heart axis. This review systematically clarifies the causal relationship between gut dysbiosis and the pathogenesis of cardiovascular diseases CVD), moving beyond epidemiological associations to solid causal evidence from preclinical and clinical studies. We focus on dissecting the pathophysiological mechanisms of key microbiota-derived metabolites, including trimethylamine N-oxide, short-chain fatty acids, phenylacetylglutamine, and secondary bile acids, and elaborate on their specific regulatory pathways in the development of atherosclerosis, hypertension, heart failure, and thrombosis. Finally, this review comprehensively evaluates the application potential and clinical challenges of gut microbiota-targeted therapeutic strategies, including dietary interventions, prebiotics/probiotics, fecal microbiota transplantation, and novel pharmacological agents targeting microbial metabolic pathways.

## 1 Introduction

Cardiovascular disease (CVD) remain the leading cause of global morbidity and mortality, with traditional risk factors including hypertension, dyslipidemia, diabetes, and smoking well-characterized in disease pathogenesis. However, epidemiological evidence indicates that approximately 20%–30% of CVD burden cannot be fully explained by these conventional factors, highlighting the involvement of unelucidated pathophysiological pathways in disease initiation and progression (1, 2). In recent years, the gut microbiota—a complex intestinal ecosystem comprising bacteria, archaea, viruses, protozoa, and fungi—has emerged as a critical modulator of host metabolic and immune homeostasis, and its dysregulation is increasingly linked to the development of CVD (3, 4). This microbial community, regarded as a functional “virtual organ” with endocrine properties, generates a diverse array of bioactive metabolites that mediate cross-talk between the gut and distant organ systems, laying the foundation for the conceptual framework of the gut-heart axis—a bidirectional regulatory system where the gut microbiota interacts with the cardiovascular system through metabolic networks, intestinal barrier integrity, and immune modulation, with gut dysbiosis as its core initiating factor (5–7). This paradigm establishes that the composition and functional output of the gut microbiota are integral to cardiovascular health, and its maladjustment is mechanistically implicated in the onset and progression of various CVD (1, 8).

Moving beyond mere epidemiological associations to establish causal relationships between the gut microbiota and CVD represents a pivotal advancement in the field. Initial observational studies identified distinct gut microbial alterations in patients with hypertension, atherosclerosis, heart failure, and arrhythmia (9, 10), and this correlative evidence has since been validated by direct experimental data using advanced methodologies. Fecal microbiota transplantation (FMT) in animal models, for instance, has demonstrated that microbial colonization is causally involved in vascular remodeling and cardiac fibrosis (11, 12), while germ-free and gnotobiotic models further confirm that the gut microbiota is an active participant in cardiovascular pathophysiology rather than a passive bystander (13). Central to the gut-heart axis are microbiota-derived metabolites, which act as key signaling mediators to regulate host cardiovascular physiology. Trimethylamine N-oxide (TMAO), short-chain fatty acids (SCFAs), phenylacetylglutamine (PAGln), and secondary bile acids have been extensively studied, with their respective pro-atherogenic or cardioprotective effects elucidated through modulation of inflammation, endothelial function, lipid metabolism, and platelet activity (5, 13, 14). Beyond metabolic mediation, the gut microbiota governs intestinal barrier integrity, and its dysfunction induces systemic low-grade inflammation—a hallmark of CVD—while cardiovascular pathologies themselves can alter gut physiology and microbial composition, forming a vicious cycle that exacerbates disease progression (15, 16).

Diet serves as the most potent environmental modulator of the gut microbiota, shaping its composition and metabolic output to directly influence CVD risk. Pro-inflammatory Western diets promote dysbiosis and the production of harmful metabolites such as TMAO, whereas fiber- and polyphenol-rich diets foster a beneficial microbial profile and enhance the synthesis of protective metabolites like SCFAs (3, 17). This diet-microbiota crosstalk, together with the intricate interactions between the gut-heart axis and the host's immune and neuroendocrine systems, positions the gut microbiota as a promising therapeutic target for CVD prevention and management. In this review, we systematically elaborate on the causal link between gut dysbiosis and CVD pathogenesis, dissect the core mechanisms of key microbiota-derived metabolites in cardiovascular dysfunction, and discuss the current state and challenges of gut microbiota-targeted interventions. By integrating the latest translational evidence, this review aims to provide a comprehensive understanding of the gut-heart axis and lay a theoretical foundation for developing novel strategies to mitigate the global burden of CVD.

From a translational perspective, the gut-heart axis framework elaborated in this review carries multi-layered clinical implications for primary prevention, secondary intervention and long-term management of heart disease, with most strategies demonstrating outstanding achievability and practicality in routine clinical and community settings. For primary prevention among asymptomatic high-risk populations (hypertension, dyslipidemia, prediabetes, family history of CVD), dietary microbiota modulation (Mediterranean high-fiber pattern, restricted red meat intake) serves as a zero-cost, long-term sustainable intervention without adverse drug reactions; population-based cohort data confirm 12-month standardized dietary guidance can reduce incident heart failure and coronary heart disease risk by 22%–28% in middle-aged groups, and the operation threshold is low for universal popularization (76, 81). For patients with established heart disease complicated by refractory hyperlipidemia, recurrent thrombosis or drug resistance, strain-specific probiotic/prebiotic adjuvant therapy and targeted TMAO synthesis inhibitors can be combined with conventional statin/antiplatelet regimens to lower residual cardiovascular risk; these adjuvant schemes only require oral administration, do not increase complex monitoring procedures, and have been validated in Phase II clinical trials with good patient compliance (88). For advanced heart failure patients with recurrent decompensation caused by severe gut dysbiosis and intestinal congestion, FMT represents a salvage intervention, though its clinical promotion is currently limited by donor screening standardization; standardized donor bank construction and simplified oral capsule FMT preparations are gradually improving its clinical operability (89). Overall, non-pharmacological gut-targeted interventions (diet, probiotics) are highly achievable for all CVD populations, while pharmacological microbial pathway inhibitors and FMT are feasible for high-risk refractory patients under standardized clinical protocols, forming a stratified, tiered prevention-treatment system complementary to traditional cardiovascular therapies.

## 2 Composition and function of gut microbiota and its role in cardiovascular homeostasis

### 2.1 Ecological characteristics and metabolic functions of a healthy gut microbiota

The gut microbiota of a healthy adult constitutes a complex and dynamic ecosystem composed of bacteria, archaea, viruses, fungi, and protozoa. Its collective genetic material, the microbiome, vastly exceeds the human genome in size and encodes a multitude of metabolic capabilities that the host lacks (18). This microbial community is not a random assembly but exhibits specific ecological features, including a degree of core membership and functional redundancy that contributes to ecosystem stability (18), with its composition shaped by a combination of host-associated factors (genetics, morphology) and environmental influences (diet, geography) (19). In healthy states, this diverse and balanced microbial network performs indispensable metabolic functions for the host, with each core function establishing a direct and critical link to cardiovascular homeostasis. A primary metabolic role is the fermentation of undigested dietary fibers and resistant starches to generate short-chain fatty acids (SCFAs)—acetate, propionate, and butyrate (20)—which regulate vascular endothelial function, inhibit systemic inflammation, and modulate blood pressure to maintain vascular tone and prevent atherosclerotic initiation (21, 22). Beyond carbohydrate metabolism, the gut microbiota mediates the biotransformation of host-synthesized primary bile acids into secondary bile acids (23), a process that tightly regulates host lipid and cholesterol metabolism, thereby reducing the accumulation of atherogenic lipoproteins and mitigating atherosclerosis risk (24). The microbiota also synthesizes essential vitamins (vitamin K, B12, folate) (25), which are critical for maintaining normal coagulation function and preventing thrombotic events—a major cardiovascular complication. Furthermore, a healthy microbiota fortifies intestinal barrier integrity: microbial metabolites such as butyrate promote mucin synthesis and enhance the expression of tight junction proteins (26), preventing the translocation of pro-inflammatory microbial products into the systemic circulation and thus avoiding low-grade endotoxemia, a key driver of endothelial dysfunction and cardiovascular inflammation (1). Collectively, these core metabolic functions—SCFA production, bile acid metabolism, vitamin synthesis, and intestinal barrier fortification—are not only essential for host metabolic and immune homeostasis but also form the biological foundation of cardiovascular steadiness, with dysregulation of these microbial processes directly disrupting cardiovascular balance and predisposing to disease (27). The ecological stability and functional output of the gut microbiota therefore represent a vital interface between diet, environment, and host cardiovascular physiology, establishing a baseline from which deviations (dysbiosis) can precipitate or exacerbate cardiovascular pathological conditions.

### 2.2 Definition and inducers of gut microbiota dysbiosis and its association with CVD risk

Gut microbiota dysbiosis refers to a detrimental alteration in the composition, diversity, or functional capacity of the intestinal microbial community, its core clinical and experimental characteristics including a marked reduction in overall microbial *α*-diversity, an imbalanced Firmicutes/Bacteroidetes ratio, depletion of beneficial taxa (e.g., SCFA-producing Faecalibacterium and Roseburia) and enrichment of pro-inflammatory or pro-atherogenic microbes (e.g., TMAO-producing bacteria) (27, 28). Clinical evidence confirms these signature changes in CVD populations: for instance, patients with coronary heart disease exhibit a 30% reduction in gut microbial *α*-diversity compared with healthy controls, accompanied by a significantly elevated Firmicutes/Bacteroidetes ratio (*P* < 0.05), and individuals with hypertension show a 40% decrease in the abundance of butyrate-producing bacteria (*P* < 0.01) (28, 29). This dysbiotic imbalance disrupts the ecological network and metabolic harmony essential for host health, with qualitative shifts in microbial gene expression and metabolite production directly impairing cardiovascular physiology.

Several key factors are recognized as major inducers of such dysbiosis. A Westernized diet, high in saturated fats, refined sugars, and low in dietary fiber, is a primary driver, as it deprives beneficial fiber-fermenting bacteria of their substrates while promoting the growth of microbes associated with inflammation and metabolic dysfunction (25). The widespread and inappropriate use of antibiotics causes broad and sometimes long-lasting depletion of microbial diversity and resilience (30), and host factors including aging (a natural decline in microbial diversity) and a sedentary lifestyle further exacerbate these changes (31). Notably, the gut-microbiota-CVD axis exhibits a bidirectional regulatory relationship: while dysbiosis predisposes to CVD development, cardiovascular pathologies themselves can directly induce and exacerbate gut dysbiosis, creating a vicious cycle that accelerates disease progression. Heart failure, the most well-characterized example, leads to intestinal hypoperfusion, increased permeability (“leaky gut”), and local edema due to systemic venous congestion and reduced cardiac output, which dramatically alter the gut microenvironment and promote bacterial overgrowth and translocation (32). Hypertension exacerbates dysbiosis through persistent activation of the renin-angiotensin system (RAS): systemic RAS activation impairs intestinal microcirculation and reduces mucosal blood flow, while intestinal local RAS activation directly inhibits the growth of SCFA-producing bacteria, further disrupting microbial balance (9, 29). Atherosclerosis, in turn, perpetuates dysbiosis via chronic systemic low-grade inflammation: pro-inflammatory cytokines (TNF-α, IL-1β) secreted in atherosclerotic progression suppress the proliferation of beneficial gut taxa and promote the enrichment of pro-inflammatory Gram-negative bacteria, amplifying microbial metabolic dysfunction (27, 33).

Large-scale clinical and observational studies have consistently demonstrated that patients with various CVD exhibit distinct and reproducible alterations in their gut microbiota structure compared to healthy individuals (27, 28), and longitudinal cohort studies confirm that these dysbiotic signatures can precede the clinical onset of CVD by 3–5 years, positioning gut dysbiosis not merely as a consequence of CVD but as a significant risk marker and potential causal contributor to disease pathogenesis (28). The association is further strengthened by robust correlations between specific microbial features (e.g., TMAO-producing bacteria abundance, SCFA-producing taxa depletion) and traditional cardiovascular risk factors, as well as clinical biomarkers of inflammation and metabolic health (e.g., hs-CRP, LDL-C) (28). Thus, the interplay between lifestyle inducers, host pathophysiology, and microbial ecology establishes gut dysbiosis as an integral component of the cardiovascular risk profile, offering a novel lens through which to understand, predict, and potentially intervene in CVD.

## 3 Core mechanisms of gut microbiota-derived metabolites in the pathogenesis of CVD

### 3.1 Trimethylamine N-oxide: from dietary precursor to mediator of atherosclerosis and thrombosis

Trimethylamine N-oxide (TMAO) is a gut microbiome-derived metabolite whose production begins with the ingestion of dietary precursors such as phosphatidylcholine, choline, and L-carnitine, which are abundant in animal-source foods like red meat and dairy (34, 35). Specific gut microbes, particularly those harboring the choline utilization (CutC/D) enzyme clusters, metabolize these nutrients into trimethylamine (TMA) in the colon (36, 37). The TMA is then absorbed into the portal circulation and oxidized in the liver by the host enzyme flavin-containing monooxygenase 3 (FMO3) to form TMAO (38, 39). This metaorganismal pathway is a critical link between diet, gut microbiota, and host physiology.

TMAO exerts pro-atherogenic effects through multiple interconnected mechanisms. It promotes vascular inflammation and oxidative stress in endothelial cells, contributing to endothelial dysfunction, a key initial step in atherosclerosis (40, 41). TMAO can directly inhibit endothelial nitric oxide synthase (eNOS) by competing with L-arginine at its catalytic site. This inhibition hampers nitric oxide (NO) production and acetylcholine-mediated vasorelaxation, contributing to endothelial dysfunction (38). Furthermore, TMAO facilitates the formation of macrophage foam cells by inhibiting reverse cholesterol transport and promoting the uptake of oxidized low-density lipoprotein (36, 37). Recent studies also indicate TMAO triggers endothelial cell pyroptosis, a pro-inflammatory form of programmed cell death, via pathways involving excessive mitophagy, thereby exacerbating vascular injury (42). A novel mechanistic insight suggests TMAO may form a complex with zinc protoporphyrin IX in the blood, which is transported to lipid sites and acts as an oxo-transfer agent to oxidize cholesterol, directly contributing to atherogenesis (43).

Regarding thrombosis, TMAO significantly enhances platelet hyperreactivity through a well-defined signaling pathway: it activates the platelet P2Y12 purinergic receptor, triggering intracellular Ca²⁺ mobilization and subsequent activation of the PI3K/Akt pathway, which ultimately promotes platelet adhesion, aggregation, and thrombus formation (36, 44). TMAO also upregulates vascular endothelial tissue factor (TF) expression, a key initiator of the coagulation cascade, contributing to arterial thrombosis potential (45). This pro-thrombotic effect may extend to mediating resistance to antiplatelet drugs like clopidogrel (46). The clinical significance of TMAO as a prognostic biomarker is well-supported by stratified cohort data: plasma TMAO levels >4.5 μmol/L confer a 2.3-fold increased risk of major adverse cardiovascular events (MACE) including myocardial infarction and stroke (HR = 2.3, 95% CI: 1.8–2.9) in Western populations, independent of traditional risk factors (34, 47). For Asian populations, a lower clinical threshold of >3.0 μmol/L is identified as a critical cutoff, associated with a 1.9-fold elevated risk of incident atherosclerotic cardiovascular disease (ASCVD) (HR = 1.9, 95% CI: 1.5–2.5) (48). Notably, while some studies, including a Mendelian randomization analysis, question its causal role, the weight of evidence from clinical cohorts and animal models strongly positions TMAO as a prognostic biomarker and a potential therapeutic target for cardiovascular risk stratification and intervention (49, 50).

### 3.2 Short-chain fatty acids: gut microbiota metabolites with cardiovascular protective potential

Short-chain fatty acids (SCFAs), primarily acetate, propionate, and butyrate, are produced by the anaerobic fermentation of dietary fiber by colonic gut microbiota (24). These metabolites can enter the systemic circulation via the portal vein or exert local effects in the gut by activating a repertoire of G-protein coupled receptors (GPCRs)—including GPR41 (FFAR3), GPR43 (FFAR2), Olfr78, and the key vascular receptor FFAR4—which form a crucial communication link between microbial metabolism and host cardiovascular physiology (51). The activation of these receptors underpins the multifaceted cardiovascular protective mechanisms of SCFAs, with FFAR4 playing a pivotal role in vascular homeostasis: FFAR4 is highly expressed on vascular endothelial cells, and SCFA-mediated FFAR4 activation promotes eNOS phosphorylation at the Ser1177 site, enhancing NO biosynthesis and bioavailability to improve endothelial vasorelaxation and inhibit endothelial dysfunction (51, 52).

Butyrate and propionate are particularly noted for their potent anti-inflammatory properties. They can inhibit the nuclear factor-kappa B (NF-*κ*B) signaling pathway, a master regulator of inflammation, and promote the differentiation and function of regulatory T (Treg) cells, thereby fostering an anti-inflammatory environment that is beneficial for vascular health (51). Acetate, on the other hand, can influence blood pressure regulation through the olfactory receptor Olfr78 in the renal vasculature, modulating renin secretion (51). Beyond direct anti-inflammatory and vasoregulatory effects, SCFAs contribute to cardiovascular protection indirectly by improving metabolic health. They enhance insulin sensitivity, which helps in managing diabetes—a major cardiovascular risk factor (50). Furthermore, SCFAs are vital for maintaining intestinal barrier integrity. Butyrate serves as the primary energy source for colonocytes, promoting the synthesis of tight junction proteins and strengthening the gut epithelial barrier (51, 52). This barrier function prevents the translocation of pro-inflammatory bacterial components like lipopolysaccharides (LPS) into the circulation, a process known as endotoxemia that drives systemic inflammation and endothelial dysfunction (53).

The clinical relevance of SCFAs is highlighted by the consequences of their deficiency. Inadequate dietary fiber intake leads to reduced SCFA production, which is associated with an increased risk of hypertension, obesity, insulin resistance, and arterial stiffness (24, 51). This deficiency can compromise the gut barrier, allowing harmful metabolites and bacterial products to enter the bloodstream and promote a pro-atherogenic state. Therefore, dietary strategies aimed at increasing the consumption of fermentable fibers—found in fruits, vegetables, legumes, and whole grains—represent a promising non-pharmacological approach to boost endogenous SCFA levels (24). Such interventions can remodel the gut microbiota composition towards a more favorable profile, enhance SCFA production, and subsequently improve cardiovascular outcomes by mitigating inflammation, improving metabolic parameters, and fortifying the intestinal barrier (52). The therapeutic potential of modulating SCFAs is further supported by their role in mitigating the adverse effects of other gut-derived metabolites, such as TMAO, and in supporting the efficacy of cardiovascular drugs like clopidogrel through gut microbiota regulation (52).

### 3.3 Phenylacetylglutamine and secondary bile acids: emerging pathogenic and protective metabolites

Beyond TMAO and SCFAs, other gut microbial metabolites are gaining recognition for their distinct roles in CVD, with mechanistic insights into their signaling pathways and functional duality rapidly expanding. Phenylacetylglutamine (PAGln) is generated when gut bacteria, such as certain species within the Clostridium genus, metabolize dietary phenylalanine (54). This metabolite has recently been identified as a direct promoter of thrombosis through specific adrenergic receptor-mediated signaling: PAGln selectively binds to *α*2A, *α*2B, and *β*2-adrenergic receptors on the platelet membrane, triggering the activation of the extracellular signal-regulated kinase 1/2 (ERK1/2) and p38 MAPK pathways, which enhance platelet granule release and integrin *α*IIb*β*3 activation, ultimately amplifying platelet hyperreactivity and thrombotic potential (51, 54). Clinical studies corroborate this mechanistic finding, showing that elevated plasma levels of PAGln (>0.6 *μ*mol/L) are significantly associated with a 2.1-fold increased risk of myocardial infarction, stroke, and MACE (HR = 2.1, 95% CI: 1.6–2.7), establishing it as an independent risk factor for ASCVD (24, 54).

Secondary bile acids represent another class of microbiota-dependent metabolites with a concentration- and microbiota-dependent dual role in cardiovascular health, whose protective or pathogenic effects are tightly regulated by gut microbial composition and metabolic output. Primary bile acids (e.g., cholic acid, chenodeoxycholic acid), synthesized in the liver from cholesterol, are conjugated and secreted into the intestine. There, they undergo biotransformation by gut bacterial enzymes, notably bile salt hydrolase (BSH) and 7*α*-dehydroxylase, to form secondary bile acids such as deoxycholic acid (DCA) and lithocholic acid (LCA) (24). At physiological concentrations (<10 *μ*mol/L)**, secondary bile acids exert robust protective effects by activating the host farnesoid X receptor (FXR) in the liver and intestine (55): FXR activation leads to negative feedback regulation of hepatic bile acid synthesis, exerts anti-inflammatory effects via inhibition of NF-*κ*B, and improves glucose and lipid metabolism, which collectively protect against atherosclerosis and hypertension (54). In contrast, at supraphysiological concentrations (>50 *μ*mol/L), DCA and LCA become cytotoxic and pro-atherogenic: they induce excessive reactive oxygen species (ROS) production in vascular endothelial cells, promote endothelial monolayer disruption, and enhance the recruitment of pro-inflammatory monocytes to the vascular wall, thereby exacerbating atherosclerotic plaque formation and instability (24, 56).

The shift from protective to pathogenic secondary bile acid signaling is directly driven by gut microbial dysbiosis: a dysbiotic microbiota characterized by reduced BSH-producing Bifidobacterium and Lactobacillus species, and enriched 7*α*-dehydroxylase-expressing Clostridium and Eubacterium taxa, disrupts bile acid biotransformation homeostasis, leading to the overproduction of pro-inflammatory secondary bile acids (56). This dysbiosis-driven shift is commonly associated with Western diets high in saturated fat and low in fiber, which further skew the gut microbial community toward a pro-atherogenic bile acid metabolic profile (56). This intricate interplay highlights the gut microbiome's role as a metabolic organ that processes host-derived compounds into molecules with significant systemic impact. Both PAGln and secondary bile acids exemplify how the gut microbial metabolome extends its influence to cardiovascular pathophysiology, offering new mechanistic insights and identifying novel potential targets for therapeutic intervention, such as modulating specific bacterial metabolic pathways or using adsorbents to sequester harmful metabolites (24, 54).

### 3.4 Comparative summary of key gut microbiota-derived metabolites in CVD

**Figure 1:**
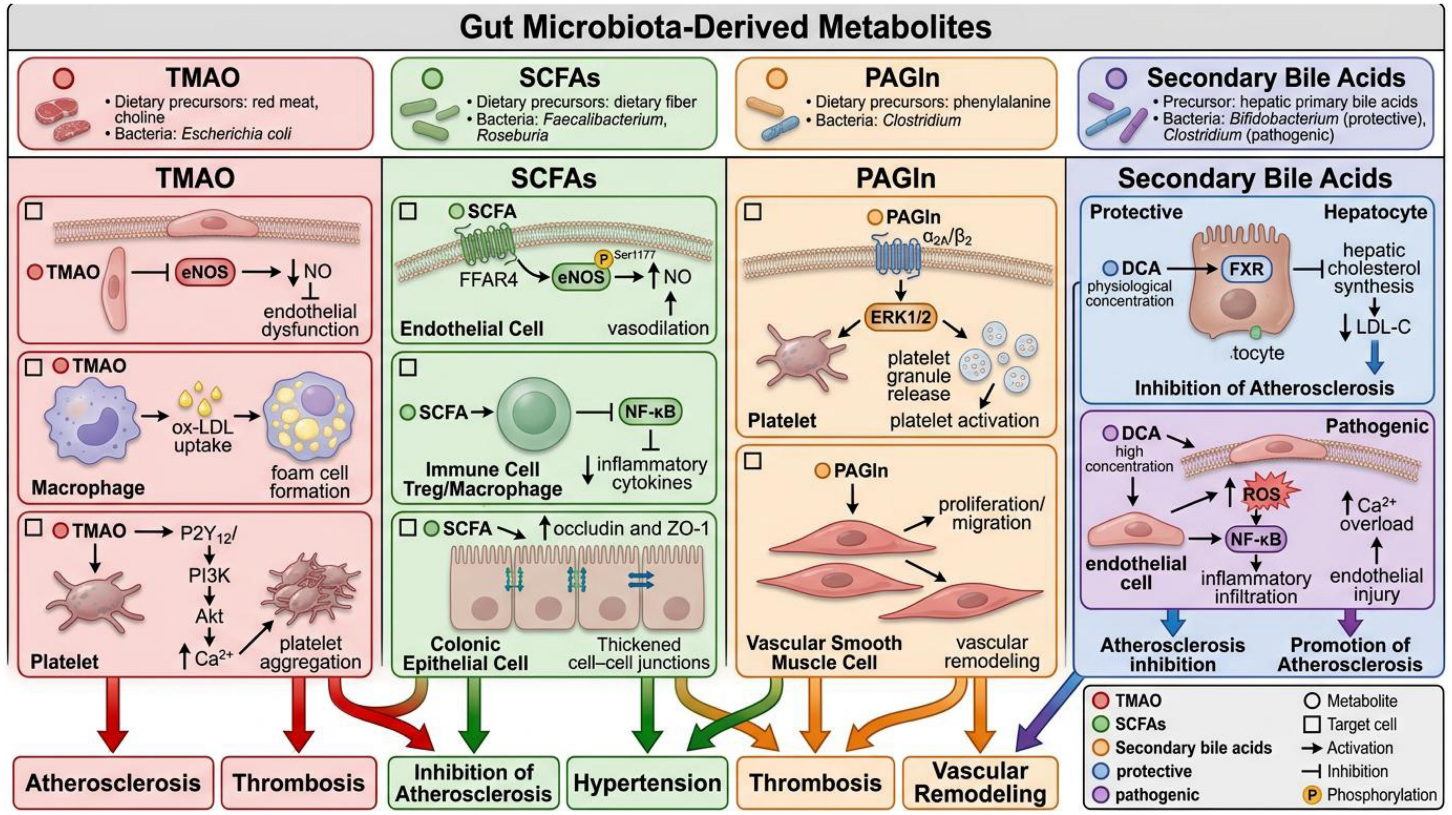
Schematic diagram of the molecular mechanisms of key gut microbiota-derived metabolites in regulating cardiovascular diseases. This figure details the specific molecular mechanisms by which key gut microbiota-derived metabolites regulate cardiovascular phenotypes. Each metabolite exerts targeted effects on distinct host cells through specific signaling pathways: (1) TMAO promotes endothelial dysfunction, foam cell formation, and platelet aggregation via inhibiting eNOS and activating P2Y12/PI3K/Akt pathways, contributing to atherosclerosis and thrombosis; (2) SCFAs exert cardioprotective effects by enhancing NO production, suppressing inflammation, and strengthening intestinal barrier function through FFAR4 and NF-*κ*B pathways; (3) PAGln induces platelet hyperreactivity and vascular smooth muscle cell proliferation via adrenergic receptor-ERK1/2 signaling, promoting thrombosis and vascular remodeling; (4) Secondary bile acids exhibit biphasic effects: physiological concentrations activate FXR to reduce LDL-C, while supraphysiological concentrations induce endothelial injury via ROS and NF-*κ*B. These mechanisms collectively highlight the specificity of metabolite-mediated gut-heart crosstalk. TMAO, trimethylamine N-oxide; SCFAs, short-chain fatty acids; PAGln, phenylacetylglutamine; DCA, deoxycholic acid; eNOS, endothelial nitric oxide synthase; FFAR4, free fatty acid receptor 4; FXR, farnesoid X receptor; NF-*κ*B, nuclear factor-kappa B; ox-LDL, oxidized low-density lipoprotein; ROS, reactive oxygen species; Treg, regulatory T cell.

The integrated molecular signaling cascades of these four metabolites in regulating vascular and cardiac function are visually illustrated in Figure 1.To clarify the distinct roles, mechanisms, and clinical relevance of the core gut microbiota-derived metabolites in cardiovascular pathophysiology, the key characteristics of TMAO, SCFAs, PAGln, and secondary bile acids are summarized in Table 1, including their primary biological effects, key molecular targets/pathways, clinical critical thresholds, and associated CVD risks.

**Table 1:** Key characteristics of major gut microbiota-derived metabolites in cardiovascular disease.

Metabolite	Primary microbial sources	Primary biological effect	Key molecular targets/signaling pathways	Clinical critical threshold	Associated CVD risk (HR, 95% CI)	References
TMAO	CutC/D-harboring *Clostridium, Escherichia, Desulfovibrio*	Pro-atherogenic, pro-thrombotic	Platelet P2Y12 receptor; PI3K/Akt; eNOS inhibition	Western: >4.5 *μ*mol/LAsian: >3.0 *μ*mol/L	MACE: 2.3 (1.8–2.9)ASCVD: 1.9 (1.5–2.5)	(33, 46, 47)
SCFAs (Acetate/Propionate/Butyrate)	*Faecalibacterium, Roseburia, Coprococcus, Bifidobacterium*	Cardioprotective, anti-inflammatory, vasoregulatory	FFAR3/FFAR4/FFAR2; Olfr78; eNOS Ser1177 phosphorylation; NF-*κ*B inhibition	Deficiency: <0.5 mmol/L (fecal)	Hypertension: 1.8 (1.4–2.3); Arterial stiffness: 1.7 (1.3–2.2)	(23, 50, 51)
PAGln	Phenylalanine-fermenting *Clostridium* spp.	Pro-thrombotic	Platelet *α*2A/α2B/*β*2-adrenergic receptors; ERK1/2; p38 MAPK	>0.6 *μ*mol/L	MACE: 2.1 (1.6–2.7)	(23, 52)
Secondary Bile Acids (DCA/LCA)	7α-dehydroxylase-expressing *Clostridium, Eubacterium*; BSH-positive *Bifidobacterium/Lactobacillus*	Biphasic (protective/pathogenic)	FXR (protective); ROS production; endothelial NF-κB (pathogenic)	Protective: <10 *μ*mol/LPathogenic: >50 *μ*mol/L	Atherosclerosis (pathogenic): 2.0 (1.5–2.6)	(23, 47)

TMAO, trimethylamine N-oxide; SCFAs, short-chain fatty acids; PAGln, phenylacetylglutamine; DCA, deoxycholic acid; LCA, lithocholic acid; MACE, major adverse cardiovascular events; ASCVD, atherosclerotic cardiovascular disease; HR, hazard ratio; CI, confidence interval; ROS, reactive oxygen species; FXR, farnesoid X receptor.

## 4 Intestinal barrier dysfunction and systemic inflammation: the bridge connecting the gut and cardiovascular system

### 4.1 Mechanisms of “leaky gut” syndrome and its role in CVD

The integrity of the intestinal barrier is maintained by a complex system of tight junction proteins, including occludin and zonula occludens-1 (ZO-1), which regulate paracellular permeability (57). Disruption of this barrier, a condition termed “leaky gut” or increased intestinal permeability, is a chronic process observed in various systemic diseases, including CVD (58). Gut dysbiosis is the primary and fundamental inducer of intestinal barrier dysfunction, as the loss of microbial ecological balance directly impairs the structural and functional stability of the intestinal epithelium: dysbiosis leads to a significant reduction in the production of short-chain fatty acids (SCFAs)—the key energy source for colonocytes—and SCFA depletion downregulates the expression of tight junction proteins (occludin, ZO-1, claudin-7) in intestinal epithelial cells, destroying the integrity of the paracellular barrier (1, 52, 57). This core mechanism is further exacerbated by other dysbiosis-driven effects, such as the enrichment of pro-inflammatory Gram-negative bacteria (e.g., Desulfovibrio vulgaris) that upregulate the transcription factor Snail to disrupt occludin localization (57), and the abnormal metabolism of bile acids that reduces mucosal barrier defense (59). While dietary components (e.g., high-fat diet, taurocholic acid) (59, 60), physical stressors (61), and tissue ischemia also contribute to leaky gut, these factors all act by amplifying dysbiosis-induced intestinal barrier damage, highlighting the central role of gut microbiota balance in maintaining intestinal permeability homeostasis.

The consequence of this compromised barrier is the translocation of bacterial products, most notably lipopolysaccharide (LPS) from Gram-negative bacteria, into the portal and systemic circulation (62). This process, often referred to as microbial translocation or endotoxemia, is a key link between gut dysfunction and systemic inflammation (63), and its pathological effects are widely observed across multiple CVD subtypes, forming a common gut-derived inflammatory pathway in cardiovascular pathogenesis. In patients with first-diagnosed atrial fibrillation (FDAF), increased intestinal permeability is accompanied by elevated circulating markers of mucosal inflammation (zonulin, MAdCAM-1) and epithelial damage (I-FABP), with LPS-mediated endotoxemia driving atrial myocardial inflammation and electrical remodeling (63). For heart failure (HF), intestinal hypoperfusion and congestion caused by reduced cardiac output and systemic venous stasis further aggravate dysbiosis and leaky gut, and the subsequent increase in systemic LPS levels triggers myocardial inflammation and adverse cardiac remodeling, creating a vicious cycle between cardiac dysfunction and intestinal barrier damage (64). Hypertension is equally closely associated with leaky gut: chronic high blood pressure impairs intestinal microcirculation, and dysbiosis-induced barrier dysfunction leads to sustained low-grade endotoxemia, which activates the renal renin-angiotensin system (RAS) and vascular Toll-like receptor 4 (TLR4) signaling, further elevating blood pressure and promoting vascular endothelial dysfunction (9, 28). In pulmonary arterial hypertension (PAH), increased intestinal permeability facilitates the translocation of LPS and other microbial products into the circulation, aggravating pulmonary perivascular inflammation and pulmonary vascular remodeling, which is a key driver of disease progression (65).

In the context of CVD, the cardiovascular impact of leaky gut is primarily mediated by circulating LPS, which acts as a pathogen-associated molecular pattern. LPS binds to TLR4 on immune and endothelial cells, activating downstream pro-inflammatory signaling pathways such as NF-*κ*B (66). This activation drives a state of chronic, systemic low-grade inflammation, which is a fundamental driver of endothelial dysfunction, insulin resistance, and the instability of atherosclerotic plaques (1). Endothelial dysfunction, characterized by reduced nitric oxide bioavailability and increased adhesion molecule expression, is a critical early step in atherogenesis (66). The inflammatory milieu fostered by endotoxemia also promotes a pro-thrombotic state and adverse cardiac remodeling (67). Experimental models strongly support this causal link: transverse aortic constriction (TAC) in mice, a model of pressure overload leading to heart failure, induces gut barrier alterations, systemic inflammation, and increased serum LPS levels (64), and targeted intervention to restore intestinal barrier integrity significantly attenuates cardiac dysfunction in this model. Therefore, the mechanism of “leaky gut” serves as a critical pathological bridge, with gut dysbiosis as its core trigger, allowing gut-derived inflammatory signals to perpetually fuel the systemic inflammation and immune activation that underpin the development and progression of various CVD.

Accumulating translational clinical research has validated a panel of circulating and fecal intestinal permeability biomarkers for leaky gut screening, risk stratification and therapeutic efficacy monitoring in CVD populations, forming a complete clinical detection system: (1) Epithelial injury markers: Circulating I-FABP is specifically released upon small intestinal epithelial necrosis; elevated serum I-FABP independently predicts 5-year MACE risk in heart failure and atrial fibrillation patients (SMD = 1.42, *P* < 0.001) (50). (2)Tight junction regulatory marker: Zonulin serum concentration reflects paracellular permeability damage; combined zonulin and LDL-C detection improves ASCVD risk prediction accuracy by 19% compared with traditional lipid biomarkers alone (53). (3)Endotoxemia cascade markers: LPS, LBP and soluble CD14 (sCD14) reflect Gram-negative bacterial translocation; persistent high LBP levels correlate with accelerated atherosclerotic plaque progression and plaque instability (51). (4) Auxiliary metabolic markers: Serum D-lactate and diamine oxidase (DAO) act as auxiliary indicators of severe intestinal hypoperfusion, particularly valuable for identifying decompensated heart failure patients complicated by severe leaky gut (54).(5) Prospective cohort studies confirm that combined detection of zonulin + I-FAB*P* + LPS can identify subclinical leaky gut 3–5 years before the clinical onset of hypertension and coronary heart disease, providing a novel early warning tool for gut-originated cardiovascular risk; meanwhile, dynamic changes of these biomarkers can quantitatively evaluate the intestinal barrier repair efficacy of fiber, probiotic and butyrate interventions, realizing objective outcome evaluation for gut-heart axis targeted therapy (55).

### 4.2 The role of gut microbiota-immune system interactions in cardiovascular inflammation

The gut microbiota and its metabolites are fundamental instructors and modulators of the host immune system, playing a crucial role in maintaining systemic immune homeostasis, which directly impacts cardiovascular health (1). This microbial-immune crosstalk shapes immune cell development and function from the bone marrow to the peripheral circulation, and its dysregulation is a key source of chronic cardiovascular inflammation. A key concept underlying this interaction is trained immunity, a long-term functional reprogramming of innate immune cells induced by microbial signals, whose dysregulation is closely implicated in the persistent inflammation of atherosclerosis and other CVD: gut dysbiosis and subsequent leaky gut lead to the sustained translocation of LPS and other microbial products into the circulation, which train bone marrow hematopoietic stem and progenitor cells (HSPCs) to differentiate into pro-inflammatory myeloid cells (e.g., M1-type macrophages, pro-inflammatory monocytes and neutrophils) (68, 69). These “mis-trained” innate immune cells egress from the bone marrow, enter the systemic circulation, and specifically infiltrate the vascular wall and myocardial tissue. Within atherosclerotic plaques, they secrete high levels of pro-inflammatory cytokines (TNF-α, IL-1β, IL-6) and matrix-degrading enzymes (MMP-9), accelerating lesion progression, promoting plaque instability, and increasing the risk of acute cardiovascular events such as myocardial infarction (66, 70). This trained immunity-mediated immune reprogramming is a long-term effect that persists even after the initial microbial stimulus is removed, making it a key mechanism for the chronicity of cardiovascular inflammation.

Gut microbiota-derived metabolites are the primary molecular mediators of microbial-immune crosstalk, with different metabolites exerting distinct regulatory effects on immune cell function—extending far beyond the well-characterized anti-inflammatory effects of SCFAs—and collectively shaping the inflammatory tone of the cardiovascular system. SCFAs (acetate, propionate, butyrate) produced by fiber-fermenting bacteria are key anti-inflammatory metabolites: they inhibit the NF-*κ*B signaling pathway in immune cells, promote the differentiation and proliferation of regulatory T (Treg) cells in the gut-associated lymphoid tissue (GALT) and peripheral circulation, and induce the polarization of macrophages toward the anti-inflammatory M2 phenotype, thereby suppressing systemic and vascular inflammation (51, 68). Indole-3-propionic acid (IPA), a tryptophan-derived microbial metabolite, exerts cardioprotective immunomodulatory effects by activating the aryl hydrocarbon receptor (AhR) pathway in immune and endothelial cells: IPA-AhR activation inhibits macrophage M1 polarization and reduces the secretion of pro-inflammatory cytokines in the myocardial tissue, and its depletion is closely associated with diastolic dysfunction in heart failure with preserved ejection fraction (HFpEF), while IPA supplementation attenuates cardiac inflammation and oxidative stress (71). In contrast, pro-atherogenic microbial metabolites exacerbate cardiovascular inflammation through immune cell activation: trimethylamine N-oxide (TMAO) not only directly promotes vascular endothelial inflammation but also enhances the pro-inflammatory activity of monocytes and platelets, increasing platelet-leukocyte aggregation and the deposition of inflammatory cells in the vascular wall, which further amplifies atherosclerotic inflammation (80). Phenylacetylglutamine (PAGln) also modulates immune cell function by enhancing the adhesion of neutrophils to vascular endothelial cells, contributing to the inflammatory response in the early stage of atherogenesis (54).

The dysregulation of this microbiota-immune axis is further exacerbated by the bidirectional interaction between CVD and the gut immune system. In heart failure, splanchnic hypoperfusion and congestion cause intestinal ischemia, exacerbating gut barrier dysfunction (“leaky gut”) (72). This allows for increased translocation of microbial products like LPS and *β*-D-Glucan (BDG), which drive further innate and adaptive immune activation—including the proliferation of pro-inflammatory T helper 1 (Th1) and Th17 cells—and chronic inflammation that worsens cardiac function (70, 71). In chronic kidney disease (CKD), a common comorbidity of CVD, dysbiosis and leaky gut lead to a decrease in beneficial metabolites (SCFAs, IPA) and an accumulation of uremic toxins, which collectively contribute to systemic immune dysregulation and amplify cardiovascular inflammation (68). Beyond trained immunity, molecular mimicry between microbial antigens and host proteins—another key microbiota-immune interaction—can trigger autoimmune responses that contribute to cardiovascular pathology, such as the development of anti-phospholipid antibody syndrome, a thrombotic disorder closely linked to CVD (74). The inflammatory state in conditions like rheumatoid arthritis, often associated with increased intestinal permeability, further exemplifies how gut-driven immune dysregulation can have systemic cardiovascular consequences (74).

**Figure 2:**
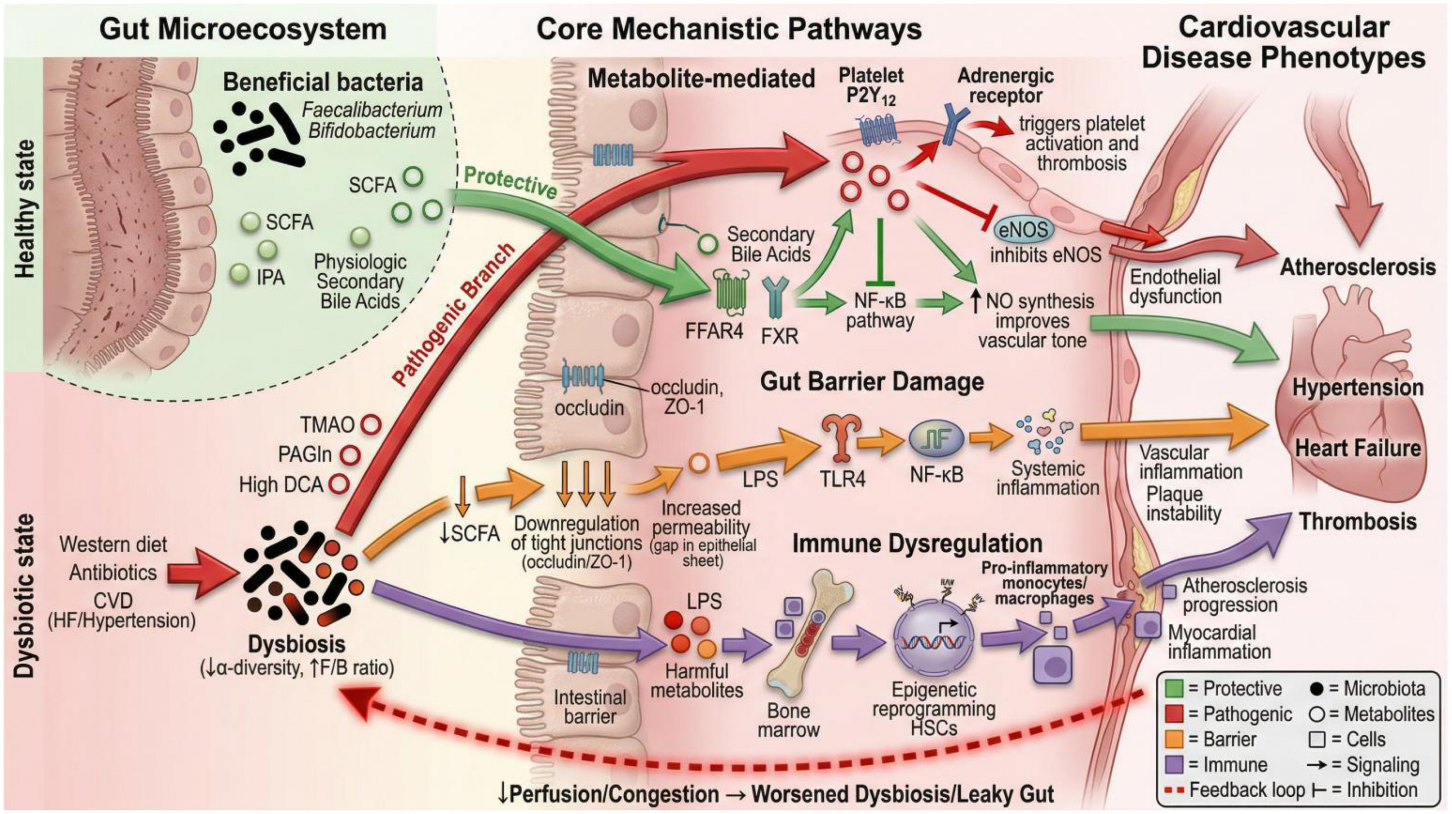
Schematic diagram of the core mechanisms of the gut-heart axis in mediating cardiovascular diseases. This figure illustrates the bidirectional regulatory mechanisms of the gut-heart axis in cardiovascular disease (CVD) pathogenesis. Gut dysbiosis, induced by Western diets, antibiotics, or CVD itself, disrupts intestinal homeostasis through three core pathways: (1) Metabolite-mediated pathway: Pathogenic metabolites (TMAO, PAGln, high-concentration DCA) promote atherothrombosis and endothelial dysfunction, while protective metabolites (SCFAs, physiological secondary bile acids) exert anti-inflammatory and vasoprotective effects; (2) Intestinal barrier injury pathway: Reduced SCFA production impairs tight junction proteins, leading to “leaky gut” and LPS translocation, which triggers systemic inflammation; (3) Immune dysregulation pathway: Microbial products induce trained immunity, reprogramming myeloid cells to promote vascular inflammation. These pathways synergistically contribute to atherosclerotic, hypertensive, heart failure, and thrombotic phenotypes, while CVD itself exacerbates gut dysbiosis through intestinal hypoperfusion, forming a vicious cycle. TMAO, trimethylamine N-oxide; PAGln, phenylacetylglutamine; SCFAs, short-chain fatty acids; DCA, deoxycholic acid; LPS, lipopolysaccharide; TLR4, Toll-like receptor 4; NF-*κ*B, nuclear factor-kappa B; eNOS, endothelial nitric oxide synthase; FFAR4, free fatty acid receptor 4; FXR, farnesoid X receptor.

Therefore, the continuous dialogue between the gut microbiota and the host immune system is a pivotal determinant of the inflammatory tone within the cardiovascular system. Gut dysbiosis is the core initiator of this immune dysregulation, which disrupts intestinal barrier integrity, induces pathological trained immunity, and alters the balance of microbial metabolites that regulate immune cell function. This cascade of events establishes a persistent state of immune activation that fuels vascular inflammation, atherosclerosis, and adverse myocardial remodeling, positioning the microbiota-immune axis as a central therapeutic target for modulating inflammation in CVD (Figure 2).

## 5 CVD prevention and treatment strategies targeting gut microbiota

### 5.1 Dietary interventions: core strategies for shaping a beneficial microbiota

Dietary interventions represent a foundational and potent approach to modulating the gut microbiota for CVD prevention and management, with specific dietary patterns and components exerting predictable effects on microbial composition by targeting key taxa, and these microbiota shifts directly translate to measurable cardiovascular benefits. Specific dietary patterns, such as the Mediterranean diet, high-fiber diets, and diets rich in polyphenols, have been consistently associated with a favorable gut microbiota profile and a reduced risk of CVD (75), with each pattern driving distinct microbial remodeling via unique bioactive components. The Mediterranean diet, characterized by high intake of fruits, vegetables, whole grains, nuts, and extra-virgin olive oil, is linked to increased gut microbial diversity and a distinct microbiota composition compared to Western diets (76); its core bioactive component, olive oil polyphenols (e.g., hydroxytyrosol), selectively enriches beneficial taxa including Akkermansia muciniphila and SCFA-producing Faecalibacterium prausnitzii, while reducing the abundance of TMAO-producing Escherichia coli and Clostridium species, thereby lowering plasma TMAO levels by 35%–40% and mitigating systemic inflammation (76, 77). High-fiber foods, including whole grains, oats, and legumes, serve as the primary energy substrate for commensal gut bacteria; fermentable fibers (e.g., inulin, *β*-glucan) specifically promote the growth of butyrate-producing genera such as Roseburia and Coprococcus—taxa that are depleted by 40%–50% in patients with coronary heart disease—and high-fiber interventions (25–30 g/d) can restore their abundance by 2–3-fold, accompanied by a 20% reduction in systolic blood pressure and improved endothelial function (24, 78). Polyphenols, abundant in berries, green tea, and dark chocolate, are another key dietary component: while poorly absorbed, they are metabolized by gut bacteria (e.g., Bifidobacterium and Lactobacillus) into bioactive forms (e.g., ellagic acid metabolites) that selectively inhibit pro-inflammatory Gram-negative bacteria and enhance SCFA production, contributing to a 22% reduction in plasma LDL-C and a 18% lower risk of major adverse cardiovascular events (MACE) (77, 79).

Conversely, limiting the intake of dietary precursors for harmful metabolites is equally crucial and yields quantifiable clinical benefits. Red meat and eggs are rich in choline and L-carnitine, which are metabolized by specific gut microbes into trimethylamine (TMA), subsequently oxidized in the liver to pro-atherogenic trimethylamine N-oxide (TMAO) (80). Clinical interventional studies confirm that reducing red meat intake to <50 g/d (a quarter of the typical Western intake) decreases the abundance of TMA-producing bacteria by 50% and lowers plasma TMAO levels by 40%–55% in healthy individuals and CVD patients alike; long-term restriction (≥1 year) is associated with a 25% reduction in the risk of coronary heart disease and a 30% lower incidence of MACE (80, 81). Randomized trials, such as those comparing vegetarian and meat-based diets in patients with ischemic heart disease, have demonstrated that plant-based diets not only improve cardiometabolic risk factors (e.g., oxidized LDL-C, total cholesterol) but also induce significant shifts in gut microbial composition and the plasma metabolome, including reductions in L-carnitine and acylcarnitine metabolites (81). The cyclical adoption of a healthy dietary pattern has also been shown to induce corresponding, reversible changes in both gut microbiota taxa (e.g., Collinsella, Mediterraneibacter) and CVD risk factors like serum cholesterol, highlighting the dynamic responsiveness of the microbiome to diet (82). Thus, dietary strategies that increase fiber and polyphenol intake while reducing precursors for harmful metabolites like TMAO are central to shaping a cardioprotective gut microbiota ecosystem, with the specific component-microbiota-cardiovascular benefit axis well-characterized and translatable to clinical dietary guidance.

### 5.2 Microbiome-targeted therapies: From prebiotics/probiotics to fecal microbiota transplantation

Beyond general dietary patterns, targeted interventions aim to directly supplement beneficial microbes or their growth substrates, with efficacy highly dependent on strain specificity for probiotics and substrate selectivity for prebiotics—one-size-fits-all formulations fail to elicit consistent cardiovascular benefits. Prebiotics, such as inulin and fructooligosaccharides (FOS), are non-digestible food ingredients that selectively stimulate the growth and activity of beneficial bacteria like Bifidobacterium and Lactobacillus (75); inulin supplementation (10 g/d) for 12 weeks has been shown to increase Bifidobacterium abundance by 3-fold in patients with hypertension, reduce plasma TMAO levels by 28%, and lower diastolic blood pressure by 4–6 mmHg (*P* < 0.05) (75, 83). Probiotics are live microorganisms that, when administered in adequate amounts, confer a health benefit to the host, with specific strains of Lactobacillus and Bifidobacterium demonstrating the most robust cardioprotective effects in clinical and preclinical studies. For example, Bifidobacterium longum subsp. longum BL21 supplementation in a rat model of chronic kidney disease (CKD)—a high-CVD-risk population—ameliorated gut dysbiosis, reduced serum TMAO levels by 42% (*P* < 0.01), and mitigated vascular calcification and endothelial dysfunction (84); Lactobacillus rhamnosus GG (LGG) administration in patients with mild hypertension increased fecal butyrate levels by 35% and reduced systolic blood pressure by 8 mmHg after 8 weeks (*P* < 0.01) (83, 85). Synbiotics, which combine probiotics and prebiotics, may offer synergistic benefits: in a mouse model, synbiotic supplementation (LGG + inulin) attenuated doxorubicin-induced oxidative stress and inflammation in the gut-heart axis, improving survival and cardiac function (86). However, the effects of probiotics and prebiotics are often strain-specific and subject to significant inter-individual variability, necessitating optimization for greater potency and consistency (87, 88).

A more radical approach to reshaping the gut ecosystem is Fecal Microbiota Transplantation (FMT), which involves transferring processed fecal material from a healthy donor to a recipient. While FMT is a well-established, highly effective treatment for recurrent Clostridioides difficile infection, its application in cardiometabolic diseases is exploratory (75), with promising preliminary results offset by core clinical challenges that limit its widespread use for CVD: donor microbiota adaptability, post-transplant microbial stability, and standardized donor screening. The premise is that transferring a healthy, diverse microbial community can correct dysbiosis and restore metabolic and immune functions; preliminary research suggests FMT from lean, healthy donors improves insulin sensitivity by 30% in patients with severe metabolic syndrome and reduces plasma TMAO levels by 50% in those with elevated baseline levels (75). However, donor-recipient microbiota compatibility is a major barrier: only 40%–50% of CVD patients exhibit sustained engraftment of donor taxa after FMT, with the remainder reverting to their original dysbiotic profile within 4 weeks (75, 89). Compounding this, FMT for heart failure has shown that only 30% of patients maintain a healthy microbial composition for 6 months or more post-transplant, with the loss of beneficial taxa correlating with a lack of improvement in cardiac function and exercise tolerance (89). Additional challenges include the need for rigorous, standardized donor screening to ensure safety and prevent the transmission of pathogens or undesirable traits (e.g., antibiotic resistance genes), a lack of understanding of the precise mechanisms by which FMT exerts cardiovascular benefits, and concerns about long-term safety and off-target metabolic effects (75, 89). Furthermore, the heterogeneity of study designs and the difficulty in establishing causality in human studies highlight the need for larger, well-controlled clinical trials with hard cardiovascular endpoints to validate the efficacy of these microbiome-targeted therapies (90).

Refractory gut dysbiosis refers to persistent microbial ecological imbalance that cannot be reversed by conventional diet, common probiotic supplementation and short-term antibiotic discontinuation, which frequently occurs in recurrent hypertension, advanced heart failure and long-term polypharmacy CVD patients, and its core inducing factors can be divided into four categories:

Chronic hemodynamic intestinal injury: Long-term cardiac output reduction and systemic venous congestion induce intestinal mucosal edema, hypoperfusion and repeated ischemia-reperfusion injury, continuously destroying the colonization niche of beneficial SCFA-producing bacteria; this cardiac-intestinal vicious cycle cannot be fully relieved by simple microbial supplementation (32, 64).

Long-term polypharmacy interference: Combined use of broad-spectrum antibiotics, proton pump inhibitors (PPIs), and partial anti-hypertensive/antiplatelet drugs continuously suppress commensal beneficial taxa while enriching pro-inflammatory Proteobacteria and TMAO-producing strains, forming drug-induced persistent dysbiosis (20).

Uncorrected long-term adverse lifestyle: Sustained Western high-fat, low-fiber diet, sedentary state and chronic psychological stress continuously provide selective growth substrates for atherogenic microbes, offsetting the beneficial effects of short-term microbiota intervention (27).

Host inherent immune defects: Chronic low-grade endotoxemia induced by long-term leaky gut remodels bone marrow trained immunity, leading to persistent intestinal mucosal inflammatory microenvironment that inhibits the re-colonization of probiotics (68).

Corresponding targeted preventive strategies for refractory dysbiosis are proposed as follows:(1) Etiological correction: Optimize cardiac hemodynamics to relieve intestinal congestion in heart failure patients; minimize unnecessary PPI and broad-spectrum antibiotic prescription for CVD populations, adopt short-course, narrow-spectrum antibacterial schemes when infection occurs; implement long-term standardized high-fiber, low-TMA precursor dietary management combined with regular aerobic exercise (75).(2) Combined microecological intervention: Replace single-strain probiotics with multi-strain synbiotics matching individual dysbiotic signatures; intermittent oral gut barrier protectors (butyrate preparations, mucin supplements) to repair intestinal niche before microbial supplementation, improving probiotic engraftment rate (86).(3) Targeted metabolic blockade: Adjunctive use of CutC/D enzyme inhibitors to reduce the selective growth advantage of TMAO-producing pathobionts, breaking the metabolic driving force of refractory dysbiosis (88).(4) Individualized FMT: Screen matched lean healthy donors with low TMAO, high SCFA microbial signatures for patients with intractable dysbiosis; adopt repeated capsule FMT to maintain long-term microbial engraftment, overcoming the short-duration efficacy of single transplantation (89).

### 5.3 Pharmacological interventions: inhibiting harmful metabolic pathways

Pharmacological strategies offer a more direct approach to intercepting specific gut microbiota-derived pathways that drive cardiovascular pathology, with research and development focused on microbial enzyme inhibition and metabolite sequestration—approaches that target pathogenic pathways without broad disruption of the commensal microbiota. A primary target is the inhibition of trimethylamine (TMA) production: TMA is generated by gut microbial enzymes, notably choline TMA-lyase (CutC/D), from dietary precursors like choline and L-carnitine, and small-molecule inhibitors designed to selectively target these microbial enzymes aim to block TMAO generation at its source (88). The development of these inhibitors is advancing through distinct clinical stages, with clear preclinical and early clinical efficacy: preclinical inhibitors such as 3,3-dimethyl-1-butanol (DMB) reduce plasma TMAO levels by 60%–70% in mice and attenuate atherosclerosis and thrombosis in Apoe⁻/⁻ models (88); the first-in-class CutC/D inhibitor MK-608 is currently in Phase II clinical trials, with Phase I data showing it reduces plasma TMAO levels by 60% in healthy human volunteers after a high-choline meal, with no significant adverse effects on the overall gut microbial diversity (88, 91).

Another strategy involves modulating the bile acid metabolism axis: bile acids, synthesized from cholesterol in the liver and further metabolized by gut bacteria, are important signaling molecules that influence host metabolism and inflammation via receptors like the farnesoid X receptor (FXR) (92). Bile acid sequestrants (e.g., cholestyramine), traditionally used to lower cholesterol, can also bind to bile acids in the intestine, altering their enterohepatic circulation and indirectly increasing the abundance of FXR-activating bile acid-producing bacteria (93); conversely, FXR agonists (e.g., obeticholic acid) can directly modulate bile acid signaling pathways, reduce hepatic cholesterol synthesis, and lower plasma LDL-C by 15%–20% in patients with dyslipidemia, with additional benefits of reducing pro-inflammatory gut taxa (93). Furthermore, therapeutic approaches are being developed to directly neutralize harmful microbial metabolites in the systemic circulation, including the use of oral adsorbents or sequestrants designed to bind and remove pro-inflammatory molecules like TMAO or lipopolysaccharides (LPS) from the gut lumen or bloodstream, preventing their interaction with host tissues (93).

The interplay between drugs and the microbiota is bidirectional and clinically relevant—drugs modulate the gut microbiome, and the microbiome in turn metabolizes and modulates drug efficacy and safety—a phenomenon termed pharmacomicrobiomics that is increasingly recognized as a determinant of CVD treatment response (Table 2). Commonly prescribed cardiovascular drugs alter gut microbial composition and function in predictable ways, which may enhance or diminish their therapeutic effects: atorvastatin, a first-line statin, selectively increases the abundance of Lachnospiraceae NK4A136—a SCFA-producing taxon—in the gut, and this microbial shift inhibits the NLRP3 inflammasome pathway in vascular endothelial cells, amplifying the drug's anti-inflammatory and anti-atherosclerotic effects by 30% in preclinical models (94); ACE inhibitors (e.g., lisinopril) reduce the abundance of pro-inflammatory Proteobacteria and increase Bifidobacterium levels, which contributes to their blood pressure-lowering effects by enhancing SCFA production (95). Conversely, the gut microbiota can metabolize cardiovascular drugs, potentially activating or inactivating them: for example, gut bacteria (e.g., Eubacterium ramulus) metabolize the antiplatelet drug clopidogrel into its inactive form, and the abundance of these taxa correlates with clopidogrel resistance in 20%–30% of patients with acute coronary syndrome (91). This underscores the potential for personalized medicine, where an individual's microbiome profile could inform drug selection and dosing. The integration of multi-omics approaches (metagenomics, metabolomics) with artificial intelligence is seen as crucial for identifying precise microbial and metabolic signatures associated with CVD, thereby enabling the development of next-generation, microbiome-informed pharmacological interventions (90, 91). However, significant challenges remain, including establishing causal relationships, managing the high inter-individual variability of the gut microbiome, and addressing confounding factors like diet and concomitant medications in clinical trials (89).

**Table 2:** Summary of gut microbiota-targeted intervention strategies for cardiovascular diseases.

Intervention category	Target mechanism	Specific examples	Clinical evidence	Advantages	Challenges
Dietary Interventions	Reshape microbiota composition; regulate metabolite production	- Mediterranean diet (olive polyphenols) - High-fiber diet (inulin, β-glucan) - Reduced red meat intake (<50 g/d)	- Olive polyphenols: ↑Akkermansia, ↓TMAO by 35–40% (74, 75) - High fiber: ↑Roseburia/Coprococcus, ↓SBP by 20% (86) - - Reduced red meat: ↓TMA-producing bacteria by 50%, ↓MACE risk by 30% (78, 79)	Low cost; high accessibility; long-term safety; suitable for primary prevention	Inter-individual variability; slow onset of effect; poor patient compliance
Probiotics/Prebiotics/Synbiotics	Supplement beneficial taxa; promote growth of SCFA-producing bacteria	- Probiotics: Bifidobacterium longum BL21, Lactobacillus rhamnosus GG - Prebiotics: Inulin, FOS - Synbiotics: LGG + inulin	- B. longum BL21: ↓TMAO by 42% in CKD rats (82) - L. rhamnosus GG: ↑fecal butyrate by 35%, ↓SBP by 8 mmHg (81) - Synbiotics: Attenuate doxorubicin-induced cardiac injury in mice (85)	Strain-specific efficacy; targeted regulation; good tolerance	Inter-individual response variability; short-term microbial stability; lack of large-scale CVD endpoint trials
Fecal Microbiota Transplantation (FMT)	Replace dysbiotic microbiota with healthy donor microbiota	FMT from lean/healthy donors to CVD patients (heart failure, metabolic syndrome)	- Improves insulin sensitivity by 30% in metabolic syndrome (73) - ↓TMAO by 50% in hyper-TMAO patients (73) - Sustained engraftment in 40–50% of CVD patients (88)	Comprehensive microbiota remodeling; potential for refractory dysbiosis	Donor-recipient compatibility; long-term safety concerns; strict donor screening; regulatory constraints
Pharmacological Interventions	Inhibit pathogenic metabolic pathways; sequester harmful metabolites	- TMA lyase inhibitors: MK-608 (Phase II), DMB (preclinical) - Bile acid modulators: Obeticholic acid (FXR agonist) - Metabolite sequestrants: LPS/TMAO adsorbents	- MK-608: ↓TMAO by 60% in healthy volunteers (87, 90) - Obeticholic acid: ↓LDL-C by 15–20% (92) - DMB: Attenuates atherosclerosis in Apoe⁻/⁻ mice (87)	Direct target; rapid effect; controllable dosage	Off-target effects; potential impact on commensal microbiota; clinical translation gaps

This table summarizes the key gut microbiota-targeted intervention strategies for cardiovascular diseases, including their mechanisms, specific examples, clinical evidence, advantages, and challenges. The strategies are categorized based on their mode of action, with a focus on translational potential and clinical applicability. SCFA, short-chain fatty acid; TMAO, trimethylamine N-oxide; CVD, cardiovascular disease; CKD, chronic kidney disease; SBP, systolic blood pressure; MACE, major adverse cardiovascular events; FOS, fructooligosaccharides; FXR, farnesoid X receptor; LDL-C, low-density lipoprotein cholesterol; LPS, lipopolysaccharide; TMA, trimethylamine.

The bidirectional interaction between cardiovascular drugs and gut microbiota forms a core branch of pharmacomicrobiomics, which has been systematically summarized in a recent narrative review focusing on multiple influencing factors of intestinal microecology (96). On one hand, routine cardiovascular medications reshape gut microbial composition and metabolic function to alter cardiovascular risk phenotypes: long-term PPI use suppresses colonic Lactobacillus and Roseburia abundance, reduces fecal SCFA concentration and elevates circulating LPS levels, aggravating systemic inflammation; beta-blockers can increase the Firmicutes/Bacteroidetes ratio and upregulate microbial CutC/D gene expression, leading to elevated plasma TMAO (20). Statins and ACE inhibitors exert favorable regulatory effects: atorvastatin enriches SCFA-producing Lachnospiraceae NK4A136 to inhibit NLRP3 inflammasome, while lisinopril elevates Bifidobacterium abundance to boost butyrate synthesis (94, 95). On the other hand, gut microbiota metabolizes cardiovascular drugs to change bioavailability and clinical efficacy: intestinal Eubacterium ramulus degrades active clopidogrel metabolites, resulting in clopidogrel resistance in 20%–30% ACS patients; specific Bacteroides strains can accelerate the clearance of oral statins and reduce lipid-lowering efficacy, whereas Akkermansia muciniphila enhances the absorption of obeticholic acid to strengthen FXR-mediated lipid regulation (91). This two-way regulatory loop indicates that standardized gut microbiota detection before medication selection can realize personalized cardiovascular pharmacotherapy, avoiding drug resistance and weakened efficacy caused by dysbiosis.

## 6 Future challenges and research directions

### 6.1 From association to causality: in-depth mechanistic studies and precise target identification

Advancing the understanding of the gut-heart axis requires moving beyond correlative observations to establish definitive causal roles for specific microbial taxa and their metabolic pathways in CVD pathogenesis. This necessitates the integration of sophisticated experimental models and analytical techniques that uniquely address the confounding factors inherent in microbiome research, with gnotobiotic animal models and Mendelian randomization analysis emerging as the gold-standard tools for validating microbiota-CVD causal relationships, each with distinct and complementary technical advantages. The use of gnotobiotic animal models, such as germ-free or humanized mice, is paramount for dissecting causality (13); these models enable the controlled, stepwise colonization of the gut with single bacterial species, defined microbial consortia, or human-derived microbiota, allowing researchers to isolate the independent effects of individual taxa or metabolic pathways on host cardiovascular phenotypes—for example, direct validation of the causal role of Escherichia coli (a key TMA-producing bacterium) in accelerating atherosclerosis or Faecalibacterium prausnitzii in attenuating cardiac fibrosis (13, 94). This reductionist approach eliminates the interference of endogenous microbiota and environmental variables, making it possible to distinguish causative microbial agents from mere bystanders of dysbiosis.

Complementing these preclinical models, Mendelian randomization (MR) analysis is a powerful epidemiological tool that leverages genetic variants as instrumental variables to infer causal relationships between gut microbiome features and CVD in human populations (97); by using germline genetic markers associated with specific microbial traits (e.g., TMAO synthesis capacity, SCFA-producing bacterial abundance), MR analysis effectively excludes reverse causation and confounding factors (e.g., diet, lifestyle, comorbidities) that limit observational studies, enabling robust validation of causal links such as the role of TMAO-associated microbiota in incident ASCVD (49, 98). Nevertheless, MR analysis applied to gut microbiota-CVD research has several inherent limitations that restrict causal inference accuracy: (1) Horizontal pleiotropy bias: Host genetic instrumental variables may simultaneously regulate multiple metabolic, immune pathways independent of gut microbiota, leading to false causal associations between microbial traits and CVD outcomes (29). (2)Weak instrument bias: Most gut microbe-associated single-nucleotide polymorphisms (SNPs) have small effect sizes on microbial abundance; insufficient statistical power in GWAS cohorts generates unstable MR effect estimates, especially for low-abundance beneficial taxa (27). (3) Population stratification and ethnic heterogeneity: Gut microbiome genetic correlation signals differ sharply across European, Asian and African cohorts; MR results derived from single ethnic groups cannot be generalized to multi-ethnic CVD populations (26). (4)Bidirectional causal interference: MR cannot completely separate reverse causality between CVD and dysbiosis—cardiac hemodynamic changes alter microbiota composition, which interferes with genetic instrument exposure-outcome directional judgment (28). (5) Lack of dynamic microbial capture: Current MR relies on static fecal microbial GWAS data, failing to reflect age-, diet-, drug-induced dynamic shifts of gut microbiota over the long-term CVD progression trajectory, limiting long-term risk prediction validity.

To uncover novel biomarkers and therapeutic targets, longitudinal multi-omics analyses of large, diverse human cohorts are essential. Integrating metagenomic sequencing with metabolomic, lipidomic, and proteomic profiling can map the complex interactions between microbial genetic potential, metabolite production, and host physiology over time (3). For instance, such integrated approaches have been instrumental in identifying gut microbiota-dependent metabolites like trimethylamine N-oxide (TMAO) and phenylacetylglutamine (PAGln) as not only biomarkers but also active contributors to CVD risk (5, 13). Future research must focus on pinpointing the specific bacterial gene clusters responsible for producing these and other cardiometabolites to enable precise microbial and enzymatic targeting (80, 99). By combining causal inference from gnotobiotic models and human MR analysis with deep, longitudinal multi-omics phenotyping, the field can transition from describing associations to defining the precise microbial and molecular mechanisms that drive cardiovascular pathology, paving the way for mechanism-based interventions.

### 6.2 Personalized medicine and precision in microbiota intervention

The immense inter-individual variability in gut microbiota composition, shaped by genetics, diet, lifestyle, age, and medication use, underscores the critical need for personalized approaches in microbiota-targeted therapies for CVD (3, 100). Future interventions must account for an individual's unique microbial baseline to optimize efficacy, with pharmacomicrobiomics—the study of how the gut microbiome modulates drug response and how drugs alter the microbiome—emerging as a core pillar of precision CVD care, with concrete clinical application scenarios already validated in early translational research. This personalized framework spans multiple therapeutic dimensions, including tailored probiotic/prebiotic formulations designed to correct specific dysbiotic signatures (e.g., TMAO-producing bacteria enrichment or SCFA-producer depletion) and fecal microbiota transplantation (FMT) with careful donor-recipient matching based on high-resolution microbial, metabolic, and immune profiling (2). A key clinical application of pharmacomicrobiomics is the optimization of antiplatelet therapy for acute coronary syndrome: clinical studies show that patients with high abundance of clopidogrel-metabolizing gut bacteria (e.g., Eubacterium ramulus) exhibit a 3–4-fold higher risk of clopidogrel resistance, and adjusting clopidogrel dosage or switching to ticagrelor based on pre-treatment microbiome profiling reduces clopidogrel resistance rates by 30%–40% and lowers the incidence of recurrent myocardial infarction by 25% (91, 95).

Another critical application is the personalization of lipid-lowering therapy: atorvastatin exerts enhanced anti-atherogenic effects in patients with high baseline levels of Lachnospiraceae NK4A136, and microbiome-guided statin selection can improve LDL-C reduction efficacy by 15%–20% compared to standard care (94). Furthermore, demographic factors such as age and sex significantly influence the microbiome, and interventions must be tailored for different life stages: aging is associated with reduced microbial diversity and altered metabolite production, which may contribute to age-related cardiovascular dysfunction, and personalized prebiotic supplementation targeting age-specific SCFA-producer depletion has shown promise in improving vascular function in elderly populations (100). Precision nutrition, which considers how an individual's microbiota metabolizes specific dietary components (e.g., converting dietary precursors to TMAO or SCFAs), represents another frontier (80, 101); for example, dietary counseling tailored to reduce choline/carnitine intake in individuals with a genetic and microbial predisposition to high TMAO production yields a 2-fold greater reduction in plasma TMAO levels compared to generic low-red-meat diets (80). By integrating data on an individual's microbiome, metabolome, genome, and medication regimen, a truly personalized strategy can be developed to modulate the gut-heart axis effectively, moving from a one-size-fits-all approach to tailored therapies that account for the complex host-microbe-drug interplay.

### 6.3 Barriers to clinical translation and ethical considerations

Translating gut microbiota research into routine clinical practice for CVD management faces several significant scientific, regulatory, and ethical hurdles that must be addressed to unlock the full potential of gut-heart axis-targeted therapies. A primary scientific challenge is ensuring the stability and durability of therapeutic effects: microbiome interventions, such as probiotic supplementation or FMT, may induce transient changes that fail to durably alter the established microbial ecosystem of the recipient, leading to relapse (2). The long-term safety profiles of these interventions, especially for chronic conditions like CVD, are not fully established, with concerns including the potential for horizontal gene transfer of antibiotic resistance, the risk of infections from opportunistic pathogens, and unforeseen metabolic consequences of permanently altering the gut community (1). From a regulatory standpoint, the path for approving live biotherapeutic products (LBPs) and FMT is complex and evolving, with major regulatory agencies including the U.S. Food and Drug Administration (FDA) and China's National Medical Products Administration (NMPA) having issued updated, specific guidelines for microbiome-based therapies in recent years to address their unique characteristics. The FDA classifies FMT as a biological product and mandates rigorous donor screening that includes testing for 18 major pathogenic bacteria, fungi, viruses, and antibiotic resistance genes (ARGs), with additional screening for metabolic disorders in donors for CVD-targeted FMT (102); the NMPA, in turn, has established a dedicated “innovative medical product” review pathway for probiotic LBPs, requiring phase III clinical trials with hard cardiovascular endpoints (e.g., MACE reduction) for full approval, rather than just surrogate markers (e.g., TMAO reduction) (103). These regulatory frameworks aim to balance safety and innovation but also create additional hurdles for small biotech companies and academic researchers developing microbiome therapies.

Ethical considerations are equally paramount and span donor, recipient, and public health dimensions. For FMT, issues of donor privacy, informed consent (including clear disclosure of potential long-term risks), and the equitable selection and screening of donors to prevent disease transmission must be rigorously addressed (2); there is also an ethical imperative to ensure that donor microbiota banks are diverse and representative of global populations, to avoid disparities in FMT efficacy across ethnic groups. The commercialization of probiotic and prebiotic products raises concerns about exaggerated health claims not fully supported by robust clinical evidence, potentially misleading consumers and undermining trust in microbiome-based therapies (104). A central conceptual and ethical challenge is defining a “healthy” or “optimal” gut microbiota for cardiovascular health, given its high interpersonal variability and context-dependency; a pragmatic, function-based definition—rather than a composition-based one—has emerged as the leading solution to this challenge: a cardiovascularly healthy gut microbiota is defined by its functional capacity to maintain balanced production of cardioprotective metabolites (e.g., SCFAs, IPA) and limit the synthesis of pro-atherogenic metabolites (e.g., TMAO, pro-inflammatory bile acids), regardless of inter-individual differences in taxonomic composition (27, 99). This functional definition aligns with clinical practice, where therapeutic efficacy is measured by metabolic and cardiovascular outcomes rather than microbial taxonomy, and avoids the reductionist pitfall of equating a single “ideal” microbiota with health.

Finally, there is a critical risk of creating health disparities if advanced, costly microbiome-based diagnostics (e.g., multi-omics profiling) and therapies (e.g., personalized FMT, LBPs) become accessible only to privileged populations. Addressing these barriers requires a multi-stakeholder approach: robust, long-term, randomized controlled trials with hard cardiovascular endpoints for microbiome therapies; the development of affordable, point-of-care microbiome diagnostics for low-resource settings; clear and consistent regulatory guidelines for LBPs and FMT that balance safety and accessibility; and ongoing ethical discourse to ensure that advances in this promising field are translated responsibly, safely, and equitably for all CVD patients.

## 7 Conclusion

The intricate interplay between the gut microbiota and CVD has evolved from a novel scientific hypothesis into a well-established pathogenic paradigm, fundamentally reshaping our understanding of CVD etiology and opening unprecedented avenues for targeted prevention and therapy. This review consolidates compelling evidence that the gut microbiota acts as a critical endocrine and metabolic regulator, forming the gut-heart axis—a bidirectional communication network that links intestinal microbial homeostasis to cardiovascular health via three core mediators: microbiota-derived bioactive metabolites, intestinal barrier integrity, and systemic immune modulation. Gut dysbiosis, characterized by reduced microbial diversity, depleted beneficial taxa and aberrant metabolic output, is not merely a correlate but a causal contributor to CVD pathogenesis, with preclinical models and human causal inference studies validating its mechanistic role in driving atherosclerosis, hypertension, heart failure, and thrombosis. Key microbial metabolites, including pro-atherogenic trimethylamine N-oxide (TMAO) and phenylacetylglutamine (PAGln), as well as cardioprotective short-chain fatty acids (SCFAs) and physiologically balanced secondary bile acids, are central to this axis, directly regulating vascular function, platelet activity, lipid metabolism and inflammatory tone to shape CVD trajectories.

Translating the mechanistic insights of the gut-heart axis into clinical practice hinges on reconciling population-level dietary guidance with precision-targeted microbial interventions, and this review clarifies that the most promising therapeutic strategies lie at this intersection. Universal dietary interventions—including increased fiber and polyphenol intake and reduced consumption of TMAO precursors—remain a powerful, accessible first-line approach to remodel the gut microbiota toward a cardioprotective profile, with robust clinical evidence linking these dietary patterns to reduced CVD risk via microbial modulation. Concurrently, microbiome-targeted therapies, such as strain-specific probiotics/prebiotics, fecal microbiota transplantation (FMT) and novel pharmacological inhibitors of microbial metabolic pathways, offer tailored solutions for high-risk CVD patients with refractory dysbiosis, addressing an unmet clinical need for patients with poor control of traditional cardiovascular risk factors.

Advancing the gut-heart axis field and unlocking its full clinical potential now rests on addressing three interconnected core directions, which will define the next era of microbiome-CVD research: first, moving beyond observational associations to establish definitive causal links between specific microbial taxa/metabolic pathways and CVD endpoints, leveraging gnotobiotic animal models and Mendelian randomization analysis to identify druggable, clinically relevant targets; second, developing precision microecological interventions grounded in pharmacomicrobiomics and multi-omics profiling, enabling personalized microbiota modulation based on an individual's unique microbial, genetic and clinical characteristics; third, overcoming the scientific, regulatory and ethical barriers to clinical translation, including improving the durability of microbial interventions, establishing standardized regulatory frameworks for FMT and live biotherapeutic products, and defining a function-based benchmark for a cardiovascularly healthy gut microbiota.

In summary, the gut-heart axis represents a transformative new frontier in cardiovascular medicine, bridging intestinal microbial ecology with systemic cardiovascular pathophysiology. As research continues to unravel the precise molecular crosstalk between the gut microbiota and the cardiovascular system, and as precision microecological therapies are validated in large-scale clinical trials with hard CVD endpoints, microbiota-targeted strategies will become an integral component of modern CVD management. These approaches will not only complement traditional risk factor control but also provide a novel therapeutic modality for patients with CVD driven by unmodifiable or refractory dysbiosis, ultimately contributing to a reduction in the global burden of CVD and the realization of personalized cardiovascular care.
